# Complications of peritonsillar abscess

**DOI:** 10.1186/s12941-020-00375-x

**Published:** 2020-07-30

**Authors:** Tejs Ehlers Klug, Thomas Greve, Malene Hentze

**Affiliations:** 1 Department of Otorhinolaryngology, Head and Neck Surgery, Aarhus University Hospital, Palle Juul-Jensens Boulevard 99 Aarhus N, Aarhus, 8200 Denmark; 2 Department of Clinical Microbiology, Aarhus University Hospital, Aarhus, Denmark

**Keywords:** Peritonsillar abscess, Complications, Bacteria, Microbiology

## Abstract

**Background:**

The vast majority of patients with peritonsillar abscess (PTA) recover uneventfully on abscess drainage and antibiotic therapy. However, occasionally patient´s condition deteriorates as the infection spread in the upper airway mucosa, through cervical tissues, or hematogenously. The bacterial etiology of PTA is unclarified and the preferred antimicrobial regimen remains controversial. The current narrative review was carried out with an aim to (1) describe the spectrum of complications previously recognized in patients with peritonsillar abscess (PTA), (2) describe the bacterial findings in PTA-associated complications, and (3) describe the time relation between PTA and complications.

**Methods:**

Systematic searches in the Medline and EMBASE databases were conducted and data on cases with PTA and one or more complications were elicited.

**Results:**

Seventeen different complications of PTA were reported. The most frequently described complications were descending mediastinitis (n = 113), para- and retropharyngeal abscess (n = 96), necrotizing fasciitis (n = 38), and Lemierre´s syndrome (n = 35). Males constituted 70% of cases and 49% of patients were > 40 years of age. The overall mortality rate was 10%. The most prevalent bacteria were viridans group streptococci (n = 41, 25%), beta-hemolytic streptococci (n = 32, 20%), *F. necrophorum* (n = 21, 13%), *S. aureus* (n = 18, 11%), *Prevotella species* (n = 17, 10%), and *Bacteroides species* (n = 14, 9%). Simultaneous diagnosis of PTA and complication was more common (59%) than development of complication after PTA treatment (36%) or recognition of complication prior to PTA (6%).

**Conclusion:**

Clinicians involved in the management of PTA patients should be aware of the wide range of complications, which may arise in association with PTA development. Especially males and patients  > 40 years of age seem to be at an increased risk of complicated disease. In addition to Group A streptococci and *F. necrophorum*, the current findings suggest that viridans group streptococci, *S. aureus*, *Prevotella*, and *Bacteroides* may also play occasional roles in the development of PTA as well as spread of infection. Complications occasionally develop in PTA patients, who are treated with antibiotics and surgical drainage.

## Background

Peritonsillar abscess (PTA) refers to pus collection between the tonsillar capsule and the pharyngeal constrictor muscle. The pathogenesis is unclarified, but closely related to an initial acute tonsillitis and it is likely that bacteria spread to the peritonsillar space via the salivary duct system [1]. The incidence of PTA peaks in young adult life (15–19 years: 167 per 100,000 population) [2]. A polymicrobial mixture of aerobes and anaerobes is commonly recovered from pus aspirates, but there is only substantial evidence to suggest pathogenic importance of Group A streptococci and *F. necrophorum* [3–5]. These two pathogens are commonly recovered in less than 50% of cases and it seems obvious that more pathogens are involved in PTA development, but the plethora of different bacteria found in an area with heavy bacterial colonization, makes it difficult to pinpoint the pathogenic bacteria from insignificant bystanders [5]. Therefore, the significant pathogens are unclear in the majority of PTA cases.

Treatment of PTA consists of surgical drainage and antimicrobial therapy. There are three accepted methods of drainage: needle aspiration, incision, and acute tonsillectomy. All three methods carry advantages and limitations [5]. Mirroring the unclarified bacterial etiology, the preferred antibiotics vary between centers and multiple regimens have been reported in recent literature [6–8].

Most likely, the vast majority of PTA patients recover uneventfully on abscess drainage and antibiotic therapy. However, the condition of PTA patients occasionally deteriorates as the infection spread in the upper airway mucosa, through cervical tissues, or hematogenously. It is undescribed whether patients with complicated PTA consult health care professionals before the development of complications or if they present with PTA and complication simultaneously. Hence, the proportion of PTA complications, which are potentially preventable, is unexplored.

When searching the literature, we were surprised to acknowledge that no previous attempts for providing a comprehensive review of complications to PTA have been done. Hence, little help was provided for clinicians, who encounter PTA patients with signs of further infectious spread and who requested an overview of this field.

The aims of the current review were threefold:

1. To describe the spectrum of complications previously recognized in PTA patients.
2. To describe the bacterial findings in PTA-associated complications, which may suggest pathogenic importance and be subject to increased attention.
3. To describe the time relation between PTA and complications in order to assess the proportion of complications, which may be avoidable.

## Main text

### Materials and methods

The Medline and EMBASE databases were systematically searched for studies reporting on patients with PTA and complications (see search strings in the Additional file 1: Appendix). Publications after 1980 in English, Danish, and German were considered. The last search was performed June 28, 2020. In addition, an extensive manual search using the reference lists (from articles included) was performed. The searches were conducted by the corresponding author, who also screened titles and abstracts for eligible studies. Final study selection and data extraction were done by the first and last authors. Agreement was reached by consensus. Articles were read with the aim to identify cases with PTA and one or more complications and elicit data describing these cases. No common definition of each complication entity was used, but the inclusion of each article was based on the author´s statements concerning the finding of PTA, the defined complication and the (likely) causality. The only exception from this reliance on author´s diagnosis, was in the differentiation between cervical necrotizing fasciitis (NF) and descending mediastinitis (DM), which was uniformly untouched in the publications. We categorized patients with described mediastinitis (with or without cervical necrosis) in the DM category and patients with cervical necrosis, but no mediastinitis, in the NF category. Some patients suffered multiple complications. In these cases, patients were categorized according to the complication deemed most severe (order: 1. upper airway obstruction < acute epiglottis < para- and retropharyngeal abscess < NF < DM. 2. sepsis < Lemierres syndrome (LS)). We excluded complications deemed to be caused by the surgical treatment of PTA (i.e. post-tonsillectomy hemorrhage) and pathology associated with uncomplicated PTA (i.e. velo-pharyngeal insufficiency). For this review, complication was defined as worsening in disease severity or the development of new symptoms or pathological changes, which may become widespread.

Statistical analyses (using STATA 15.1) were performed using the binomial probability test for gender distribution and the Fisher´s exact test to compare mortality rates between genders.

## Results and discussion

### Overview

**Fig. 1:**
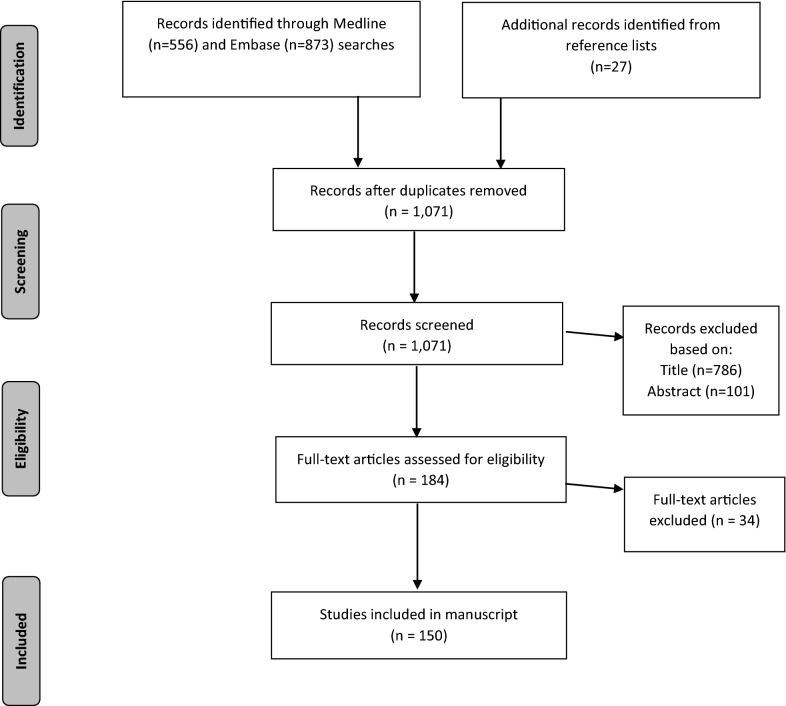
PRISMA flow diagram of the literature searches

The literature searches identified 1,047 unique records and 27 records were added from the refence lists (Fig. 1). Through title, abstract, and full-text evaluation 921 records were excluded and 150 records were included in this review.

In total, 334 patients with PTA and one or more associated complications were found in the literature. The most frequently described complications were DM (n = 113), para- and retropharyngeal abscess (n = 96), NF (n = 38) and LS (n = 35) (Table 1). There was a significant male preponderance (70%, p < 0.001, Binomial probability test) and males outnumbered females in all complication entities with more than two described cases. Information on the time relation between diagnosis of PTA and complication was available in 126 (38%) cases (Table 1). In these cases, 45 (36%) patients had PTA prior to development of complication, 74 (59%) were diagnosed with PTA and complication simultaneously, and in seven (6%) patients the complication was recognized prior to PTA diagnosis. DM and NF were the most prevalent complications among patients, who had infectious spread after initial treatment of PTA, but this time relation was also the case in other rare complications (masticator space abscess, reactive arthritis, carditis, and Kawasaki´s syndrome). The potential positive effects of aggressive treatment (i.e. acute tonsillectomy and intravenous broad-spectrum antibiotics) compared to more conservative treatment (aspiration/incision and penicillin) could not be estimated due to the limited number and heterogeneity of well-described cases.

**Table 1:** Overview of complications to peritonsillar abscesses (PTA)

Mechanism of propagation and complication	No. of patients	Males^a^	Age, median years (range)	Time relation between PTA and complication (time of diagnosis)				Acute tonsillitis prior to PTA^a^	Antibiotic treatment prior to complication^a^	Mortality^a^	Sequelae^a^
				PTA prior	Same time	PTA after	Unknown				
Upper airway spread											
Airway obstruction	14	62%	17 (0–68)	1	12	0	1	58%	88%	7%	0%
Epiglottitis	15	100%	41 (27–64)	0	5	0	10	80%	80%	0%	–
Neck tissue spread											
Para- and retropharyngeal abscess	96	77%^b^	62^c^ (14–75)	2	8	0	86	0%^d^	50%	0%	0%
Necrotizing fasciitis	38	76%^e^	51 (20–87)	17	6	0	15	22%	89%	21%	46%
Mediastinitis	113	65%^f^	52 (15–82)	13	13	0	87	41%	74%	19%	38%
Internal carotid artery lesion	7	57%	15 (3–95)	2	5	0	0	33%	50%	0%	50%
Parotid gland abscess	1	0%	60	0	1	0	0	–	100%	0%	0%
Mastricator space abscess	2	50%	83 (75–90)	2	0	0	0	0%	100%	0%	–
Hematogenous spread											
Lemierre´s syndrome	35	52%	21 (0–65)	2	21	3	9	40%	27%	10%	50%
Sepsis	2	50%	10 (0–20)	0	0	2	0	50%	100%	0%	0%
Lung infections	2	100%	38 (14–61)	1	0	1	0	50%	100%	0%	0%
Carditis	3	100%	52 (17–80)	2	0	1	0	50%	50%	67%	–
Brain abscess	1	0%	9	0	1	0	0	100%	0%	0%	100%
Septic arthritis	1	100%	55	0	1	0	0	100%	100%	0%	100%
Immunologic reaction											
Reactive arthritis	1	0%	32	1	0	0	0	100%	100%	0%	0%
STSS	1	100%	68	0	1	0	0	0%	0%	0%	0%
Unknown											
Kawasakis syndrome	2	100%	7 (7–7)	2	0	0	0	100%	100%	0%	0%
All	334	70%^2^	41 (0-95)	45	74	7	208	42%	69%	10%	21%

*PTA* peritonsillar abscess. *STSS* Streptococcal toxic shock syndrome

^a^Percentage of cases with information provided (gender: n = 235; acute tonsillitis prior to PTA: n = 98; antibiotic treatment prior to complication: n = 156; mortality: n = 273; sequelae: n = 87)

^b^p < 0.001, binomial probability test

^c^n = 5

^d^n = 4

^e^p = 0.003, binomial probability test

^f^p = 0.04, binomial probability test

**Fig. 2:**
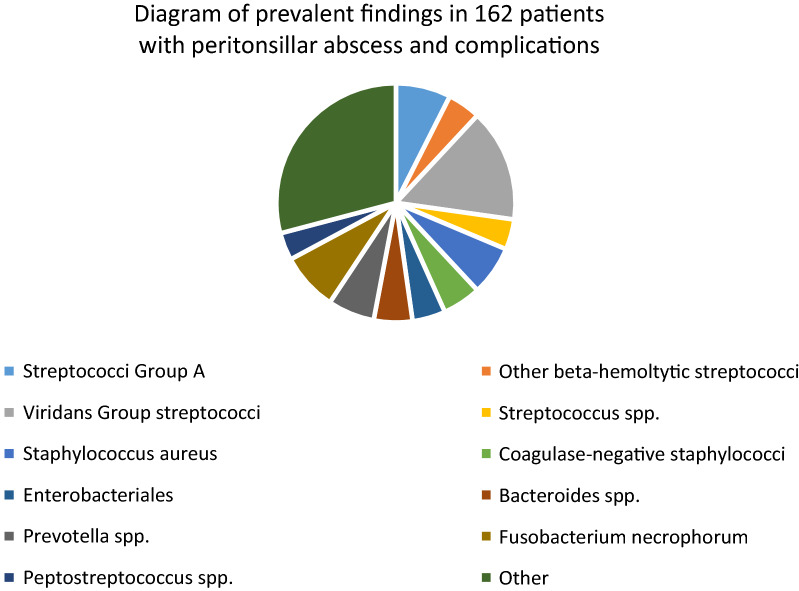
Diagram of the prevalent findings in 162 patients with peritonsillar abscess and complications

The overall mortality rate was 10% and without significant difference between genders (p = 0.77, Fishers exact test). With the caveat that information were only available in 29% and 46% of cases, respectively, acute tonsillitis prior to PTA development and antibiotic treatment prior to development of complication were prevalent (42% and 69%, respectively). These findings suggest that varying standard antibiotic regimens were insufficient to prevent the development or progression of infectious spread. Information regarding microbiologic findings were provided in 162 (49%) patients (Table 2). The most prevalent bacteria were viridans group streptococci (VGS) (n = 41, 25%), beta-hemolytic streptococci (n = 32, 20%, especially Group A (n = 20, 12%)), *F. necrophorum* (n = 21, 13%), *S. aureus* (n = 18, 11%), *Prevotella species* (n = 17, 10%), and *Bacteroides species* (n = 14, 9%). (Figure 2). In addition to Group A streptococci and *F. necrophorum*, the current findings suggest that VGS, *S. aureus*, *Prevotella*, and *Bacteroides* may also play an occasional role in the development of PTA as well as spread of infection. However, a plethora of potential pathogens, both aerobes and anaerobes, were recovered from PTA pus, sites of complication, blood, or unspecified sites (unfortunately the sites for culture were undescribed in the majority of cases). Hence, our findings support previous studies that PTAs and complications of PTA can be caused by multiple pathogens and infections are often polymicrobial (48%, Table 2). While some of the prevalent pathogens were almost exclusively found in one or two complication entities (*S. aureus*: DM; *F. necrophorum*: LS), streptococci were more evenly distributed among complications with the exception of LS (Table 2).

**Table 2:** Microbiologic findings in 162 patients with peritonsillar abscess and complications

Organisms	PPA n = 36	LS n = 26	NF n = 33	DM n = 46	Other n = 21	All n = 162
Aerobic bacteria						
Streptococcus						
Group A	9		6	1	4	20
Group C	2		3		2	7
Beta-hemolytic			1	1	2	5
Viridans group	10	2	10	15	4	41
spp.		1	2	8		11
*Enterococcus spp.*			2		1	3
*Staphylococcus aureus*	1	2	1	14		18
Coagulase-negative		1	6	6	1	14
spp.			4			4
*Haemophilus spp.*	1			1	2	4
*Enterobacteriales*			6	4	2	12
*Pseudomonas aeruginosa*		1	1	6		8
*Burkholderia cepacia*				1		1
S*tenotrophomonas maltophilia*			1			1
*Corynebacterium spp*.			1	1		2
*Eikenella corrodens*				1		1
*Arcano. haemolyticum*		1				1
*Neisseria spp.*		1				1
*Acinetobacter coloaceticus*				2		2
*Moraxella catarrhalis*				1		1
Anaerobic bacteria						
*Bacteroides spp.*		1	4	9		14
*Porphyromonas spp.*		2				2
*Prevotella spp.*	1	2	6	5	3	17
*Fusobacterium*						
*nucleatum*		2	1	1		4
*necrophorum*	3	15		1	2	21
spp.				1		1
*Peptostreptococcus spp.*		1	5	4		10
*Peptoniphilus asaccharolyticus*		1		1	1	3
*Parvimonas micra*			1			1
G + , non-spore-forming bacilli			2	3		5
Unspecified anaerobes	9		2	4	1	16
Unspecified bacteria			3	7		10
Fungi						
*Candida albicans*	1		3	2		6
*Aspergillus fumigatus*				1		1
Polymicrobial	½	4/26	18/33	33/46	5/21	61/128

Bacterial names are reported in contemporary nomenclature (in contrast to Tables 4 and 5)

The names of the following bacteria are merged

Viridans group streptococci: viridans group (n = 16), Group F (n = 1), non-hemolytic (n = 4), mitis group (n = 2), *sanguis* (n = 1), *salivarius* (n = 2), *intermedius* (n = 1), *constellatus* (n = 3), anginosus group (n = 4), and milleri group (n = 7)

*Enterococcus spp.: E. faecalis* (n = 1) and Enterococcus species (n = 2)

Coagulase-negative staphylococci: *hominis* (n = 1), *epidermidis* (n = 6), *haemolyticus* (n = 2), and coagulase-negative staphylococci (n = 5)

*Haemophilus spp.: Haemophilus influenza* (n = 3) and *Haemophilus parainfluenzae* (n = 1)

*Enterobacterales*: *Escherichia coli* (n = 2), *Enterobacter cloacae* (n = 3), Enterobacter species (n = 1), *Klebsiella pneumoniae* (n = 2), Klebsiella species (n = 1), *Proteus mirabilis* (n = 1), *Serratia marcescens* (n = 1), and *Citrobacter diversus* (n = 1)

*Bacteroides spp*.: *fragilis* (n = 8), and species (n = 6)

*Prevotella spp*.: *intermedia* (n = 1), *buccae* (n = 1), *oralis* (n = 3), *corporis* (n = 1), *melaninogenica* (n = 6), and species (n = 5)

*Peptostreptococcus spp.: Peptostreptococcus anaerobius* (n = 3) and *Peptostreptococcus species* (n = 7)

G + , non-spore-forming bacilli: *Eggerthella lenta* (n = 2*), Eubacterium species* (n = 1), *Bifidobacterium dentium* (n = 1), and *Eggerthia catenaformis* (n = 1)

Unspecified: Gram-negative rod (n = 4), Gram-negative (n = 1), Gram-positive coccus (n = 2), Gram-positive rod (n = 1), Gram-positive (n = 2)

*PPA* parapharyngeal abscess, *LS* Lemierre´s syndrome, *NF* necrotizing fasciitis, *DM* descending mediastinitis, *Spp* species (no further specification was provided in the study), *G-* Gram-negative, *G + * Gram-positive, *Arcano Arcanobacterium*

### Spread of infection in the upper airways

#### Upper airway obstruction

Upper airway obstruction may develop because of large abscess volume(s), tonsillar hypertrophy, and/or collateral mucosal edema or phlegmon of the pharynx and larynx. Eleven case reports described a total of twelve patients with PTA and upper airway obstruction without additional abscesses, epiglottitis, DM, or NF [9–19]. Patients characteristics, their treatment, and microbial findings are presented in Table 3. The majority of patients were diagnosed with (58%) and treated for (50%) acute tonsillitis prior to PTA diagnosis (mean 2.7 days). Streptococci were recovered from six of seven patients from whom bacterial cultures were performed. The site of upper airway obstruction was the oropharynx in 83% of cases and developed after PTA treatment (incision and drainage) in only one case (8%). Acute airway management was performed in four patients and one (8%) patient died before reaching hospital. No other sequelae were described. The antibiotic regimens were different from each other in all eight cases with this information provided. In addition to the twelve well-described cases, Hsiao et al. and Menezes et al. each reported a case of upper airway obstruction among 56 and 71 children with PTA, respectively [20, 21].

**Table 3:** Characteristics of patients (n = 12) with peritonsillar abscess (PTA) and upper airway obstruction

Males	7 (58%)
Children (< 16 years)	5 (42%)
Infectious mononucleosis	2 (17%)
Duration of admission (mean days)	6.1
Site of obstruction	
Oropharynx	10 (83%)
Hypopharynx/larynx	2 (17%)
Airway management	
Intubation	2 (17%)
Acute tracheotomy	2 (17%)
Surgical treatment	
Acute tonsillectomy	8 (67%)
Incision and drainage	3 (25%)
Bacterial findings in PTA cultures (n = 7)	
Streptococcus Group A	2 (29%)
Streptococcus Group C/G	2 (29%)
Viridans group streptococci	2 (29%)
*H. influenzae*	1 (14%)

#### Acute epiglottitis

Five patients with concomitant PTA and acute epiglottitis have been described in two papers [22, 23]. All patients were adult males (age range 27–64 years) and four patients were treated with antibiotics because of acute tonsillitis prior to epiglottitis and PTA diagnoses. None of the patients underwent surgical treatment of the PTA and, hence, no PTA culture results were reported. All patients recovered on different regimens of broad-spectrum antibiotics. In addition to the five well-described cases, Brandow reported four cases of concomitant epiglottitis among 156 PTA patients undergoing acute tonsillectomy [24]. Hafidh et al. described 10 adult cases of acute epiglottitis and found one patient with concurrent PTA [25]. Lastly, five cases of “epiglottis involvement” among 45 PTA patients with concomitant para- or retopharyngeal abscess were reported by Monobe et al. [26].

### Spread of infection through neck tissues

#### Para- and retropharyngeal abscess/phlegmon

Infections laterally or posteriorly to the pharyngeal constrictor muscle are referred to as para- and retropharyngeal abscess or phlegmon. These infections may arise after spread of bacteria from the teeth or upper airway mucosa through neck tissues or lymph ducts [27, 28]. Less frequently, para- and retropharyngeal infections are described as extensions of PTA [20, 26, 29–37]. In a study of 99 PTA patients by Kawabata et al., thirteen patients had spread of infection into the para- and/or retropharyngeal spaces [36]. Two pediatric studies reported concurrent PTA and para- or retropharyngeal abscess in 2/249 and 2/56 patients, respectively [20, 29]. In the latter study, all patients were treated with antibiotics only [20]. Monobe et al. stratified 45 patients with PTA and either para- or retropharyngeal abscess into superior (n = 24) and inferior types (n = 21) according to the location of the PTA [26]. The majority of superior PTAs were aspirated or incised while inferior PTAs underwent intraoral drainage (n = 14) or no surgery (n = 10). Page et al. included 31 patients undergoing immediate tonsillectomy because of either PTA (n = 13), parapharyngeal abscess (n = 13), or concomitant PTA and parapharyngeal abscess (n = 5) [30]. Côrte et al. reported four cases of PTA progressing to parapharyngeal abscess (n = 2) or retropharyngeal abscess (n = 2) [34]. Similarly, Ohori et al. described three cases of PTA and concurrent parapharyngeal abscess (one case had an additional submandibular space abscess) [35].

Information on bacterial findings were only included in three studies [31, 32, 35]. In a study of 63 patients with parapharyngeal abscess, cultures were performed in 32 of 33 patients with concomitant PTA [32]. The most common findings were Group A streptococci (29%), VGS (18%), *F. necrophorum* (12%), and Group C/G streptococci (12%). Treatment included acute tonsillectomy (33/33) and internal incision (32/33). Ohori et al. also advocated for acute tonsillectomy and internal incision based on the outcomes of three patients with concomitant PTA and parapharyngeal abscess [35]. Bacterial examinations showed *Streptococcus constellatus* + *Prevotella melaninogenica*, no growth, and *S. constellatus*, respectively. A 24-years old woman with Group A streptococci in blood cultures were surgically treated for bilateral PTA with extension into the right parapharyngeal space 1 day after undergoing PTA needle aspiration [31]. Except one patient in the case series by Ohori et al., who refused acute tonsillectomy and subsequently developed submandibular space abscess, all other studies reported that patients recovered without further spread of infection. Hence, multiple management regimes seemed to clear the infection (surgically and antibiotically).

#### Necrotizing fasciitis (NF)

NF is a rapidly progressing infection that typically involves necrosis of the superficial tissue layers but may extend to the deep fat and fascial layers [38]. The most common primary sites of infection in cervical NF are the teeth, the tonsils, and traumatic or surgical wounds [39, 40]. In a previous review of cervical NF (n = 13), Boninsegna et al. found a higher mortality rate in PTA-associated NF (31%) compared to NF of other origins (20%) and that patients treated with tonsillectomy had a better prognosis (0% mortality rate) compared to cases treated without tonsillectomy (44% mortality rate) (p = 0.056) [41]. In total, 38 cases (median age 51 years) of PTA-associated cervical NF were found (Table 1) [37, 41–66]. Significantly more males (26 cases) than females (8 cases) were described (p = 0.003, Binomial probability test). A wide variety of bacteria were found with streptococci and staphylococci being the most prevalent strains (Table 4). Multiple bacterial species were recovered in 55% (18/33) of cultures. Great variations in treatment modalities were found among the 17 patients, who were diagnosed with PTA prior to NF development, both concerning the surgery performed and choices of antibiotics (Table 4). The mortality rate was 12% (2/17) in this group compared to 50% (3/6) in the group of patients, who were diagnosed with PTA and NF simultaneously, and 18% (2/11) in patients with no information regarding this time relation. The number of patients are too small to draw conclusions concerning the effects of different treatment modalities on the risk of death.

**Table 4:** Characteristics of 34 well-described patients with peritonsillar abscess (PTA) and necrotizing fasciitis (NF)

References	Age (years)	Gender	Microbiologic findings	Treatment of PTA prior to NF development		Outcome
				Surgery	Antibiotics	
Wills [42]	35	M	*S. maltophilia*; VGS; *B. melaninogenicus*	Spon perf	Iv. pen	Survived
Wenig [43]	50	M	AHS; *F. nucleatum*; *E. lentum; B. melaninogenicus*; *P. anaerobius*; *E. cloacae*; *S. epidermidis*; Klebsiella spp.; *P. aeruginosa*	Simultaneous^a^		Died
Wenig [63]	50	M	AHS; *E. coli*; mitis group strep.; *S. san*-*guis*; *S. faecalis*; *B. fragilis; B. mela*-*ninogenicus*; Corynebacterium spp.	Incision	Iv. cefazolin	Died
Pedersen [37]	67	M	No cultures	Simultaneous^a^		Died
Lalwani [44]	33	M	GCS; *B. melaninogenicus*	Incision	Im. pen	Survived
Tovi [43]	20	M	*Enterobacter spp.; P. mirabilis*; *K. pneumoniae*	NI^b^		Died
Scott [46]	51	M	Milleri group strep; NHS; anaerobes	Drainage	Iv. cefo, fluclo, metro	Survived
Maisel [50]	29	M	VGS	NI^b^		Survived
Jackson [47]	63	M	*E. lentum*; *P. anaerobius*; *B. fragilis*	Drainage	None	Survived
Greinwald [45]	65	M	GAS; *S. epidermidis*	Incision	Iv. ampicillin	Survived
Hadfield [48]	53	M	GCS; *Bacteroides spp.*	Incision	Iv. cefo, metro	Survived
Skitalic [51]	50	F	*S. epidermidis*; (GAS antibodies)	Simultaneous^a^		Survived
Djupesland [52]	39	M	GAS	Simultaneous^a^		Died
Djupesland [52]	36	M	Milleri group strep.; *Bacteroides spp.*	Asp	Iv. pen, tobra, metro	Survived
Nielsen [58]	NI	NI	GCS	NI^b^		Died
Safak [49]	43	F	GAS; CNS; *Enterococci*	Drainage	Iv. pen, im. chlor	Survived
Goldenberg [53]	53	F	BHS	Incision	Iv. pen → pip-tazo	Survived
Vaid [61]	55	M	GAS	Simultaneous^a^		Survived
Beninsegna [41]	58	M	*S. epidermidis*	Acute tons	Iv. ampicillin, metro	Survived
Beninsegna [41]	80	M	GAS; *Peptostreptococcus spp*.; *C. albicans*	Incision	Iv. ampicillin, metro	Survived
Losanoff [54]	54	M	NHS; *Peptostreptococcus spp.*	No	Iv. ceftriaxo-ne, amoxicillin	Survived
Bono [55]	60	M	No cultures	Spon perf	Iv. ceftriaxo-rine, metro	Died
Wolf [57]	62	M	GAS	NI^b^		Survived
Wolf [57]	49	M	NHS	NI^b^		Survived
Andres [56]	87	F	*Staphylococci*	NI^b^		Survived
Andres [56]	64	F	*Staphylococci*; Gram-negative rod	NI^b^		Survived
Andres [56]	44	M	*Staphylococci*; Gram-negative rod; *C. albicans*	NI^b^		Survived
Andres [56]	44	M	*Staphylococci*; Gram-negative rod; *C. albicans*	NI^b^		Survived
Horváth [59]	55	M	No bacterial growth	NI^b^		Survived
Horváth [59]	61	F	*Parvimonas micra*	NI^b^		Survived
Flores [62]	38	M	*S. constellatus, Peptostreptococcus spp., Prevotella spp.*	Simultaneous^a^		Survived
Brown [64]	56	M	*Prevotella spp*; CNS	Incision	Clindamycin	Survived
Irani [66]	33	M	*S. aureus*	Incision	Iv. antibiotics	Survived
Burstin [65]	47	F	Milleri group strep.; anaerobes	No	Iv. antibiotics	Survived

Bacterial names are written as reported by author (and not changed according to current nomenclature)

*VGS* viridans group streptococci, *Spon perf* spontaneous perforation, *Pen penicillin*, *Iv* Intravenous, *Strep streptococci, Im* intramuscular, *Cefo* cefotaxime, *Fuclo* flucloxacillin, *AHS* alpha-hemolytic streptococci, *GCS* Group C streptococci, *NHS* non-hemolytic streptococci, *CNS* coagulase-negative staphylococci, *BHS* beta-hemolytic streptococci, *GAS* Group A streptococci, *Asp* aspiration, Tobra tobramycin, *Chlor* chloramphenicol, *Pip-tazo* piperacillin-tazobactam, *Tons* tonsillectomy

^a^Simultaneous diagnosis and treatment of PTA and NF

^b^No information regarding the time relation between PTA and NF

#### Descending mediastinitis (DM)

Cervical infections originating from PTAs may descend through the cervical planes to the mediastinum. Pneumonia, pleural effusion and empyema, shock, acute respiratory distress syndrome (ARDS), disseminated intravascular coagulation (DIC), and other complications are frequently present in association with DM.

We found 38 studies reporting a total of 113 patients with PTA-associated DM [67–104]. The median age was 52 years and there was a significant male preponderance (65%, 34/52, p = 0.04, Binomial probability test). The mortality rate was 19%. Forty-four cases were well-described and are presented in Table 5. A wide variety of bacteria were recovered from PTA pus, cervical tissues, mediastinum, or blood (Tables 2 and 5). Like PTA-associated NF, great variations in treatment modalities were found among the 13 patients, who were diagnosed with PTA prior to DM development (Table 5). No difference in mortality rates (both 8%, 1/13) was found between patients diagnosed with PTA prior to DM development and patients with simultaneous PTA and NF, but the mortality was 28% (5/18) among patients with no information concerning this time relation. From the limited number of patients, we are unable to estimate if aggressive eradication of the peritonsillar infection or broad-spectrum antibiotics reduce the risk of DM or improve the outcome compared to less comprehensive treatment.

**Table 5:** Characteristics of 44 well-described patients with peritonsillar abscess (PTA) and descending mediastinitis (DM)

References	Age (years)	Gender	Microbiologic findings	Treatment of PTA prior to DM development		Outcome
				Surgery	Antibiotics	
Zgheib [89]	52	M	Group A streptococci, *E. corrodens*	Drain	Ery → Clinda	Survived
Civen [74]	45	M	*C. diversus,* Group F streptococci, VGS, CNS, *P. bucca, P. intermedia, F. nucleatum, A. odontolyticus, B. dentium, L. catenaforme, P. anaerobius, P. asaccharolyticus*	Simultaneous^a^		Survived
Alsoub [75]	32	M	VGS	Incision	Cephalothin	Survived
Alsoub [75]	47	M	Negative cultures	Incision	Cephalothin	Survived
Cordero [88]	27	M	*S. anginosus*, anaerobes	Incision	None	Survived
Nielsen [91]	25	M	*S. anginosus, Bacteroides spp.*	Acute tons	Iv. pen + metro	Survived
Endo [93]	67	M	*Peptostreptococcus sp*., *C. albicans*, Gram-positive coccus	Simultaneous^a^		Survived
Endo [93]	63	F	Gram-positive coccus + Gram-positive rod + Gram-negative rod	Simultaneous^a^		Survived
Manecke [96]	29	M	Beta-hemolytic streptococci, *Pepto*-*streptococcus spp., Bacteroides spp.*	Simultaneous^a^		Survived
Corsten [99]	71	M	Gram-positive bacilli	No	Clinda	Survived
Sancho [76]	19	F	*A. coloaceticus, P. aeruginosa, B. fragilis*	NI^b^		Survived
Sancho [76]	71	M	*S. aureus, E. cloacae, B. fragilis*	NI^b^		Died
Sancho [76]	39	M	*S. aureus, P. aeruginosa, B. fragilis*	NI^b^		Survived
Laxmipathi [90]	70	M	*S. aureus*	Drain	NI	Survived
Asrar [97]	36	M	Non-hemolytic streptococci	Incision	Iv. Unspec	Survived
Lautermann [77]	52	NI	AHS, CNS	Simultaneous^a^		Survived
Lautermann [77]	61	NI	Negative cultures	Simultaneous^a^		Survived
Mihos [78]	26	M	*S. hominis, Fusobacterium spp.*	Drain	Unspec	Survived
Collin [79]	48	M	*S. salivarius*	Incision	Amox-clav + metro	Survived
Sandner [80]	26	M	AHS*, N. catarrhalis, C. albicans*	Simultaneous^1^		Survived
Sandner [80]	45	M	AHS, VGS, *S. aureus, S. marcescens*	Drain	Unspec	Died
Roccia [68]	65	M	*S. anginosus*	NI^b^		Survived
Roccia [68]	50	M	*S. epidermidis, B. melaninogenicus*	NI^b^		Died
Roccia [68]	53	M	Negative cultures	NI^b^		Died
Roccia [68]	20	M	*S. aureus*	NI^b^		Survived
Roccia [68]	37	F	*S. aureus*	NI^b^		Survived
Roccia [68]	16	F	*S. anginosus*	NI^b^		Survived
Roccia [68]	58	M	*S. aureus, A. fumigatus*	NI^b^		Survived
Roccia [68]	52	M	VGS, *Prevotella oralis*	NI^b^		Died
Roccia [68]	33	F	*H. influenzae*	NI^b^		Survived
Roccia [68]	65	M	*S. aureus*	NI^b^		Survived
Roccia [68]	66	M	*S. epidermidis, P. aeruginosa*	NI^b^		Survived
Roccia [68]	57	M	*S. haemolyticus, B. cepacia*	NI^b^		Died
Roccia [68]	58	F	*S. aureus*	NI^b^		Survived
Roccia [68]	53	M	VGS, *P. aeruginosa*	NI^b^		Survived
Kinzer [81]	67	NI	*S. intermedius*, *Bacteroides spp.*	Simultaneous^a^		Died
De Freitas [83]	18	F	NI	Simultaneous^a^		Survived
De Freitas [83]	30	F	NI	Incision	Unspec	Survived
Nakamura [100]	77	F	*Bacteroides spp., Corynebacterium spp.*	Simultaneous^a^		Survived
Anderson [102]	NI	F	VGS	Simultaneous^a^		Survived
Iyer [94]	74	M	Gram-positive, Gram-negative, anaerobes	Simultaneous^a^		Survived
Gallo [98]	57	M	*S. aureus* (MR)	NI^b^		Survived
Wahab [95]	29	F	Negative cultures	Asp	Pen + metro → clari + metro	Survived
Geerts [82]	34	M	*S. aureus, S. salivarius, F. necrophorum*	Simultaneous^a^		Survived

Bacterial names are written as reported by author (and not changed according to current nomenclature)

*M* male, *F* female, *Drain* drainage, *Ery* erythromycin, → changed to, *Clinda* clindamycin, *VGS* Viridans group streptococci, *CNS* Coagulase-negative staphylococci, *Tons* tonsillectomy, *Iv* intraveneous, *Pen* penicillin, *Metro* metronidazole, *NI* no information, *Unspec* unspecified, *AHS* alpha-hemolytic streptococci, *Amox-clav* amoxicillin-clavulanate, *MR* Methicillin resistant, *Asp*: aspiration, *Clari* clarithromycin

^a^Simultaneous diagnosis and treatment of PTA and DM

^b^No information regarding the time relation between PTA and DM

#### Internal carotid artery lesion

Bacteria may spread to the internal carotid artery from a PTA resulting in vascular necrosis [105, 106] or (pseudo-)aneurysm [107–111]. PTA-associated carotid artery lesions were occasionally reported in the preantibiotic era [106], but we only found seven described cases within the last 40 years [105–111]. In addition to symptoms of PTA, some patients presented with intermittent severe hemorrhage (n = 2) and anisocoria (n = 1) [105, 106]. In two cases, a bleeding pseudoaneurysm developed 35–45 days after PTA drainage [107, 109], while the PTA and the carotid artery lesion were diagnosed simultaneously in five cases [105, 106, 108, 110, 111]. Four patients were children (3–15 years of age) and two patients were elderly (65 and 95 years of age, respectively). The bacterial findings in PTA specimens were only described in two cases [109, 111]: streptococcus Group A (n = 1) and VGS (n = 1). Ligation or embolization of the internal carotid artery was carried out in all but one patient (who underwent stenting) [108]. All patients survived, but sequelae were described in three cases: Horners syndrome (n = 3) and discrete contralateral neurological deficit (n = 1) [106, 109, 110].

#### Parotid gland abscess

Reiss and Reiss described a 60-year-old female, who was diagnosed with concomitant bilateral PTA and unilateral parotid gland abscess [112]. PTA cultures grew *Klebsiella pneumonia*e. The patient recovered after acute tonsillectomy, external cervical incision, and ciprofloxacin therapy.

#### Masticator space abscess

Hidaka et al. described two cases of PTA and masticator space abscess in a 75-year-old man and a 90-year-old woman [113]. Both patients recovered without sequelae after intensive antibiotic therapy, incision of the PTA, and drainage of the masticator space abscess. PTA pus cultures grew *Prevotella corporis *+* Gemella morbillorum* and *peptostreptococcus species*, respectively.

### Hematogenous spread of infection

#### Lemierre´s syndrome (LS)

The sequence of events leading from (peri-)tonsillar infection to LS is not fully understood. A key step is spread of bacteria from the tonsil to the internal jugular vein, which could be hematogenous via the tonsillar veins or direct spread. According to Riordan, histological observations in the pre-antibiotic era showed abscesses in the proximity of the tonsil in patients with LS, and bacteria were thought to spread from these abscesses deeper into the loose connective tissue of the pharynx and attach to the walls of the veins [114].

In total, our literature search identified 35 described cases (median age 21 years) of PTA and LS [115–142]. Ten patients (40%) were diagnosed with acute tonsillitis and seven patients were treated with antibiotics for a mean of 4.8 days prior to PTA and LS development (Table 1). The time relation between PTA and LS was described in 26 cases: 21 patients were diagnosed with PTA and LS simultaneously; two patients were treated for PTA prior to LS development, and in three patients LS was diagnosed prior to PTA recognition. There was a slight male preponderance (52%, 13/25). The mortality rate was 10% (3/31) and sequelae were described in four cases. The microbiological findings were reported in 26 cases (Table 6). The bacterial findings in PTA aspirates were only reported in five cases and only two cases described coherent findings in PTA aspirates and blood cultures (*S. aureus* and *F. necrophorum*, respectively) [126, 141]. The frequent observation of preceding acute tonsillitis, the common simultaneous diagnosis of PTA and LS, and the lack of knowledge concerning the bacteria involved in the presented PTA cases, raise the question if development of LS is a complication of the initial acute tonsillitis or secondary to the PTA.

**Table 6:** Microbiological findings in 26 patients with peritonsillar abscess (PTA) and Lemierre´s syndrome

	Culture site			
Bacteria	PTA (n = 5)	Blood (n = 18)	Liver abscess (n = 1)	Unknown (n = 3)
Aerobes				
Alpha-hemolytic streptococci	1			
Viridans group streptococci		1		1
*Staphylococcus aureus*	1	1		
Coagulase-negative staphylococci	1			
*Arcanobacterium haemolyticum*		1		
*Pseudomonas aeruginosa*				1
*Neisseria spp.*				1
Anaerobes				
*Fusobacterium necrophorum*	1	14	1	
*Fusobacterium nucleatum*	1	1		
*Prevotella spp.*	2			
*Porphyromonas spp.*	1	1		
*Bacteroides spp.*				1
*Peptostreptococcus spp.*				1
*Peptoniphilus asaccharolyticus*		1		

The names of the following bacteria are merged

Viridans group streptococci: milleri group (n = 1) and *Streptococcus constellatus* (n = 1)

*Spp* species

Compared to acute tonsillitis, LS seems less frequently associated with PTA. Hagelskjaer-Kristensen et al. found two patients with LS and PTA compared to 35 patients with LS and acute tonsillitis [135]. In a study of eleven cases of LS (with oropharyngeal origin) by Rhigini et al., six patients had PTA [117]. Based on the multiple cases described in literature, most patients seem to develop LS without evidence of PTA, PPA, or other deep neck abscesses or phlegmon. However, patients with concomitant LS and PTA may be underestimated as most patients diagnosed with LS were never examined by an otorhinolaryngologist and some patients may have had smaller, undiagnosed PTAs, which subsided during the massive antibiotic treatment. In this regard, it is noteworthy that the primary causative bacteria in LS, *F. necrophorum*, can be recovered more frequently in PTA patients (up to 60% among teenagers) than patients with acute tonsillitis (12–48%) [143, 144].

#### Sepsis

We found two described cases: A 20-year-old male developed pericardial effusion, acute respiratory failure, and sepsis some days after treatment of a sore throat with azithromycin [145, 146]. Blood cultures grew *F. necrophorum*, but there were no signs of internal jugular vein thrombosis. After some days with broad-spectrum antibiotic treatment, a CT angiogram revealed a PTA, which was drained. No information on sequelae was provided. A 3-month-old girl with congenital bone marrow failure developed septic shock, but recovered after drainage of PTA and broad-spectrum antibiotic treatment [147]. No microorganisms were identified.

#### Lung infections

Two cases of PTA and lung complications without DM were described [148, 149]. A 14-year-old male was admitted with concomitant PTA, cervical phlegmon and lung abscesses [149]. *F. necrophorum* was found in cervical pus specimens. Apart from pus aspiration, no surgical intervention was performed. Nevertheless, he recovered without sequelae on antibiotics. A 61-year-old male with an undrained PTA (for 2 days) developed parapharyngeal space infection, bilateral pleural effusion, and dorsal lung collapse [148]. PTA cultures grew beta-hemolytic streptococci and anaerobic rods. He underwent unspecified surgery and received broad-spectrum antibiotics. No information regarding sequelae was provided.

#### Carditis

We found three described cases of PTA complicated by carditis. A 58-year-old male experienced spontaneous perforation of a PTA (treated with intravenous penicillin) the day after receiving amoxicillin because of progressive sore throat [150]. The following day the patient died from myocarditis. *E. coli* and Enterococci were recovered from pericardium fluid, while normal flora was found in tonsillar cultures. A 17-year-old male had cardiac arrest a few hours after quinsy tonsillectomy [151]. His death 12 days later was deemed to be caused by the pancarditis found at autopsy. No bacterial findings were reported. An 80-year-old male were treated for infectious endocarditis and PTA [152]. Blood cultures grew *S. constellatus* and *S. mitis* was recovered from the PTA.

#### Brain abscess

One case of brain abscess as complication of PTA was found: Sankararaman et al. described a 9-year-old girl with a frontal brain abscess secondary to an ipsilateral PTA [153]. The cerebral abscess was conservatively treated with (undefined) intravenous antibiotics and the patient´s condition improved in the days after drainage of the PTA (8 mL of thick pus growing *Prevotella species*). She developed hydrocephalus requiring an external ventricular drain.

#### Septic arthritis

Sever et al. reported a 55-year-old female, who developed septic arthritis of the temporo-mandibular joint and PTA after initial acute tonsillitis [154]. Cultures from both sites of infection grew *Staphylococcus haemolyticus*. The patient was treated with broad-spectrum antibiotics and PTA drainage. She recovered with an asymptomatic defect in the wall of the external ear canal.

### Immunologic reactions

#### Reactive arthritis

Mazur et al. described a 32-year-old female, who developed poststreptococcal reactive arthritis of the left ankle after initial acute tonsillitis (treated with amoxicillin-clavulate for 5 days) and PTA (treated with incision and cefuroxime + metronidazole) [155]. PTA cultures grew Group A streptococci, *Prevotella oralis*, and *Haemophilus parainfluenzae*. The patient recovered within 4weeks.

#### Streptococcal toxic shock syndrome

One case of Streptococcal toxic shock syndrome complicating a PTA was described [156]: a 68-year-old male developed coagulation disturbances, hypotension, and renal function impairment secondary to a Group A streptococcus-positive PTA. The patient was treated with acute tonsillectomy and broad-spectrum antibiotics. He recovered without sequelae.

### Unknown mechanism

#### Kawasaki disease

Kawasaki disease is a potentially fatal pediatric illness characterized by fever, rash, conjunctivitis, cervical lymphadenopathy, stomatitis, myocarditis, and coronary artery vasculitis. The etiology of the disease is unknown and the diagnosis is established by the recognition of five out of six characteristic clinical criteria. Two cases of PTA and Kawasaki disease was found [157, 158]. A 7-year-old boy developed PTA 4 days after finishing a 10-days course of amoxicillin-clavulanate because of Group A streptococcus-positive acute tonsillitis [158]. After initial treatment with incision and intravenous penicillin, the patient developed increasing cervical lymphadenopathy, torticollis, rash, arthritis, acute myocarditis, pleural and pericardial effusions, and heart failure. He was treated with acute tonsillectomy, pericardiocentesis, salisylates, and gamma globulin. He recovered without sequelae. PTA cultures were negative. Another 7-year-old boy was admitted with culture-negative acute tonsillitis and treated with intravenous penicillin [157]. Five days later, PTA was suspected and 1 ml of pus was recovered at time of tonsillectomy. PTA pus cultures were negative. During the next 2 days, he developed classic symptoms of Kawasaki disease, which was treated with salisylates and gamma globulin and the patient recovered.

## Limitations

The majority of cases were described in case reports. Publication bias may influence the relative number of cases for each entity and distort the picture of complications towards more severe cases. Mortality rates could be both over- or underestimated. We were unable to estimate the prevalence of complications to PTA, but it is obviously low. The collected description of the microbiology associated with complicated PTA was limited by different (and often undeclared) culture sites and methods, different levels of species identification and the relatively limited number of cases with thorough description of the bacterial findings. Preceding acute tonsillitis was rather common among patients with complicated PTA and the exact causality of the complications were occasionally difficult to determine, but the initial acute tonsillitis underscores the intimate relationship to PTA, which has previously been questioned by a number of authors [159, 160].

## Implications for practice

It is important for clinicians to acknowledge that a wide range of complications may arise in association with PTA, either simultaneously or after PTA treatment. Complications have been described in PTA patients at all ages, but ages were closely related to complication types (e.g. the median ages were 51–52 years among patients with DM and NF compared to 21 years among patients with LS). We found a pronounced male preponderance (70%), which is significantly higher than in uncomplicated PTA (58%) [5]. The current study stresses that PTA and infectious spread may develop in patients treated with antibiotics (even if surgical drainage is performed) and highlights the importance of thorough patient information at time of discharge, especially in males and  > 40 years of age, who seems to be at increased risk of complications. Improved knowledge on risk factors for complicated disease and the significant pathogens associated with PTA development and further infectious spread are important to reduce the morbidity of throat infections.

## Conclusions

The current study provides an overview of complications of PTA described in the literature within the last 40 years. Complications after spread of bacteria through neck tissues (the major being DM, para- and retropharyngeal abscess, and NF) constituted 77% of the described cases and other mechanisms of spread were much less frequent (through airway mucosa, 9%; hematogenous, 13%; immunologic/unknown, 1%). In addition to Group A streptococci and *F. necrophorum*, the current findings suggest that VGS, *S. aureus*, *Prevotella*, and *Bacteroides* may also play occasional roles in the development of PTA as well as spread of infection. Spread of infection could potentially have been avoided in a minority of PTA cases, who presented to health care professionals for the treatment of PTA prior to developing complications. However, the potential for avoidance of complications was difficult to estimate and the majority of patients presented with simultaneous PTA and complication.

## **Supplementary information**

**Additional file 1**.

## Data Availability

From references. The full dataset can be obtained from the corresponding author upon request.

## Abbreviations

PTA: Peritonsillar abscess
NF: Necrotizing fasciitis
DM: Descending mediastinitis
LS: Lemierres syndrome
VGS: Viridans group streptococci
