# Supplementary material for: Complications of peritonsillar abscess
Source: Ann Clin Microbiol Antimicrob. 2020 Jul 30;19:32. doi: 10.1186/s12941-020-00375-x (PMC7391705; doi:10.1186/s12941-020-00375-x)
Supplement: Supplementary file 1 — Additional file 1. [file 12941_2020_375_MOESM1_ESM.docx]

**Appendix**

| Results of searches conducted in the Medline and EMBASE datases, June 28, 2020 | Database | |
| --- | --- | --- |
| Search strings | Medline | Embase |
| (”peritonsillar abscess” OR quinsy) AND (complication*) | 463 | 572 |
| (”peritonsillar abscess” OR quinsy) AND mediastinitis | 67 | 77 |
| (”peritonsillar abscess” OR quinsy) AND (“lemierre’s syndrome” OR “lemierre syndrome”) | 38 | 90 |
| (”peritonsillar abscess” OR quinsy) AND “necrotizing fasciitis” | 34 | 50 |
| (”peritonsillar abscess” OR quinsy) AND “parapharyngeal abscess” (Embase: “retropharyngeal abscess”) | 46 | 211 |
| (”peritonsillar abscess” OR quinsy) AND obstruction | 77 | 106 |
| (”peritonsillar abscess” OR quinsy) AND (“carotid artery” OR pseudoaneurysm) | 35 | 53 |
| (”peritonsillar abscess” OR quinsy) AND “disseminated intravascular coagulation” | 2 | 4 |
| (”peritonsillar abscess” OR quinsy) AND “brain abscess” | 7 | 21 |
| (”peritonsillar abscess” OR quinsy) AND epiglottitis | 29 | 73 |
| (”peritonsillar abscess” OR quinsy) AND (sepsis OR bacteremia) | 57 | 116 |
| ("peritonsillar abscess" OR quinsy) AND ("streptococcal toxic shock syndrome" OR "toxic shock" OR STSS) | 3 | 15 |
| ("peritonsillar abscess" OR quinsy) AND ("pneumonia") | 21 | 114 |
| ("peritonsillar abscess" OR quinsy) AND ("ARDS" OR "acute respiratory distress syndrome") | 2 | 4 |
| ("peritonsillar abscess" OR quinsy) AND ("jugular vein thrombosis") | 9 | 20 |
| ”peritonsillar abscess” AND complication* AND (microbiology OR bacteria) | 136 | 79 |
| ”peritonsillar abscess” AND complication* AND (pathogenesis OR pathogen) | 329 | 43 |
| (”peritonsillar abscess” OR quinsy) AND endocarditis | 7 | 14 |
| ("peritonsillar abscess" OR quinsy) AND ("adult respiratory distress syndrome" OR “acute respiratory distress syndrome”) | 4 | 6 |
| "Peritonsillar Abscess/complications"[Mesh] AND "Lemierre Syndrome"[Mesh] | 4 |  |
| "Peritonsillar Abscess/complications"[Mesh] AND "Fasciitis, Necrotizing"[Mesh] | 11 |  |
| " Peritonsillar Abscess/complications"[Mesh] AND " Mediastinitis "[Mesh] | 24 |  |
| All combined | 556 | 873 |
